# MAD2L2 Dimerization Is Not Essential for Mitotic Regulation

**DOI:** 10.3390/ijms252111485

**Published:** 2024-10-25

**Authors:** Nomi Barda, Philippa Jennifer Ayiku, Amit Bar-on, Sahar Movshovitz, Tamar Listovsky

**Affiliations:** 1 Molecular Biology Department, Ariel University, Ariel 40700, Israel; neomipe@ariel.ac.il (N.B.); philippaj@ariel.ac.il (P.J.A.); amit.baron@msmail.ariel.ac.il (A.B.-o.); saharm@ariel.ac.il (S.M.); 2 Adelson School of Medicine, Ariel University, Ariel 40700, Israel

**Keywords:** MAD2L2, mitosis, TLS

## Abstract

MAD2L2 is a small HORMA domain protein that plays a crucial role in DNA repair and mitosis. In both TLS and shieldin, the dimerization of MAD2L2 via its HORMA domain is critical for the stability and function of these complexes. However, in mitosis, the dimerization state of MAD2L2 remains unknown. To assess the importance of MAD2L2’s dimerization during mitosis, we utilized CRISPR/Cas9 to generate MAD2L2 knockout cells, which were subsequently complemented with MAD2L2 species carrying different dimer-disrupting point mutations. We assessed the ability of these MAD2L2 dimer-disrupting mutants to regulate mitosis by evaluating early mitotic events and mitotic fidelity. Our findings indicate that MAD2L2 can function in its monomeric form during mitosis, suggesting that MAD2L2 homodimerization is dispensable for early mitotic regulation. Furthermore, our results suggest that the binding of CDH1 to MAD2L2 is a key regulating factor in mitosis that may actively prevent the formation of MAD2L2 dimers, thereby shifting the cellular balance toward MAD2L2-CDH1 interaction. Thus, the equilibrium between the monomeric and dimeric forms of MAD2L2 is an important cellular factor regulating the MAD2L2-containing complexes.

## 1. Introduction

Genome integrity, chromatin regulation, and mitotic progression are vital functions in the living cell [1,2]. MAD2L2 (mitotic arrest-deficient 2-like 2), also known as Rev7, is a small protein containing a HORMA (Hop1p, Rev7p, and MAD2) domain. The HORMA domain is an evolutionarily conserved domain known for its role in protein–protein interactions and diverse functions [3]. It plays a critical role in the activity of MAD2L2, including the entrapment of interacting proteins and peptides, dimerization, and stabilization of protein complexes. These activities primarily rely on conformational changes in MAD2L2, transitioning from an inactive open (O) conformation to an active closed (C) conformation. These conformational changes regulate the formation of diverse complexes, which include various proteins specific to different cellular processes [4,5,6].

MAD2L2 was initially discovered in the translesion synthesis (TLS) pathway, an evolutionarily conserved mechanism that enables DNA damage tolerance by facilitating DNA replication and single-strand gap-filling opposite to DNA lesions, without repairing the damage. TLS is activated in response to DNA damage, and it is considered a mutagenic pathway as TLS polymerases lack proofreading. Therefore, TLS access to DNA is highly regulated. Mechanistically, TLS is divided into a two-step process in which usually a Y-family polymerase replicates through the lesion, followed by extension of the distorted DNA by Pol zeta (ζ). The Pol ζ core complex contains the polymerase catalytic subunit Rev3 and MAD2L2, which stabilizes the polymerase and then facilitates interaction with Rev1 polymerase [7,8,9] and the processivity-enhancing subunits [10]. In vivo, the activity of Pol ζ in TLS depends on the formation of a core complex consisting of Rev3, MAD2L2 homodimer, and Rev1 [11,12]. While the atomic structure of the MAD2L2 homodimer in TLS remains unsolved, its complex composition and regulation have been elucidated through biochemical, biophysical, and genetic approaches [13,14,15]. Dysfunctional TLS leads to the accumulation of various types of DNA damage, which can ultimately result in cell death [16,17,18].

Beyond TLS, MAD2L2 participates in various complexes in diverse pathways. It has been shown to cooperate with 53BP1 in mediating non-homologous end joining (NHEJ) and preventing homologous recombination (HR) [19,20]. There, MAD2L2 forms a four-subunit single-stranded DNA-binding complex, consisting of MAD2L2, SHLD1, SHLD2, and SHLD3, named the shieldin complex [21,22,23]. Within the shieldin complex, MAD2L2 exists as a homodimer, presenting a different homodimerization conformation from TLS. The homodimerization of MAD2L2 is critical for stabilizing SHLD3 for proper complex formation [6,24,25].

Another intriguing function of MAD2L2 is its function as an inhibitor of the anaphase-promoting complex/cyclosome (APC/C) [26,27]. The APC/C is an essential mitotic E3 ubiquitin ligase protein complex, responsible for progression through mitosis and G1 and regulating critical events such as the metaphase–anaphase transition, mitotic exit, and G1 phase control [28,29,30]. The APC/C has two activator proteins, CDC20 and CDH1(FZR1), both of which are themselves APC/C substrates [31,32,33]. MAD2L2 inhibits premature APC/C activation by sequestering CDH1 from the APC/C and supporting the timely activator switch of the APC/C [34]. Premature APC/C activation disrupts proper mitotic regulation, resulting in accelerated substrate degradation, faster mitotic progression, and the appearance of severe mitotic defects like anaphase bridges and lagging chromosomes. Interestingly, CDH1 does not carry the canonical “φφxPxxxPP” motif typically observed in numerous MAD2L2-binding partners that interact at the MAD2L2’s seat belt loop [4,5,14]. However, CDH1 binds to MAD2L2 at the “Rev1-binding” motif, utilizing residue L186 [35], which is also used by SHLD3 [6]. Additionally, CDH1 utilizes its KxLx motif, important for the CDH1-APC/C interaction, to bind to MAD2L2 [35]. We explored the importance of MAD2L2 dimerization for its mitotic role, and here, we present data revealing that MAD2L2 homodimerization is not essential for mitotic regulation, and the main factor controlling prometaphase is the ability of MAD2L2 to properly bind to CDH1. In agreement with our data, a recent work by Vassel et al. [36] revealed that MAD2L2’s dimerization is dispensable for mitosis but not for TLS or DNA damage repair.

Understanding the structure–function of MAD2L2 and its preferential complex formation is a central goal for a better understanding of these fundamental cellular processes and their role in early tumorigenesis.

## 2. Results

### 2.1. Monomeric MAD2L2 Can Bind to CDH1

**Figure 1:**
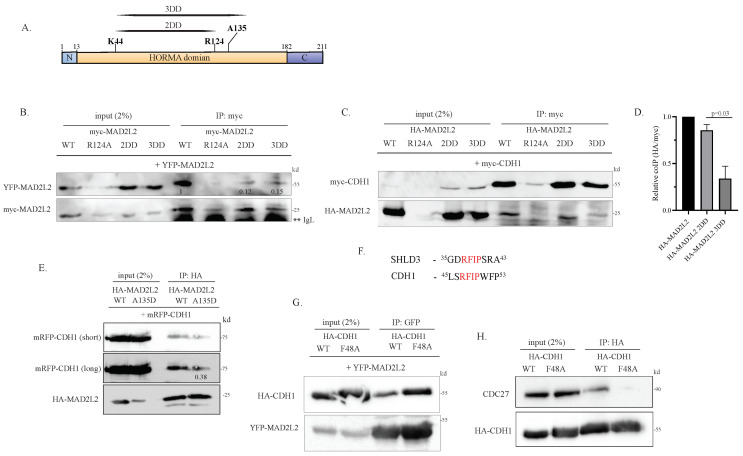
Monomeric MAD2L2 can bind to CDH1: (**A**) Schematic representation of MAD2L2 residues mutated to disrupt homodimerization. (**B**) Myc-2DD and myc-3DD mutants displayed reduced binding to YFP-MAD2L2. HEK293 cells were co-transfected with the indicated plasmids, and α-myc IP was performed to assess MAD2L2 homodimer formation. The binding of myc-MAD2L2 to YFP-MAD2L2 was reduced in all myc-MAD2L2 mutants when compared to WT. **IgL–IgG Light chain. (**C**) MAD2L2-CDH1 binding was reduced in the HA-3DD mutant. HEK293 cells were co-transfected with the indicated plasmids, and α-myc IP was performed to assess MAD2L2-CDH1 binding. The binding of myc-CDH1 to HA-MAD2L2 was reduced in HA-MAD2L2 3DD and HA-R124A mutants when compared to WT and HA-2DD. (**D**) The quantification of the relative amount of myc CDH1 bound to each HA-MAD2L2 mutant. Error bar = 1 SD. The additional blots contributing to this analysis are shown in Figure S1D. (**E**) HA-MAD2L2 A135D mutant displayed reduced binding to mRFP-CDH1. HEK293 cells were co-transfected with the indicated plasmids, and α-HA IP was performed to assess MAD2L2-CDH1 binding. The binding of HA-MAD2L2 A135D mutant to mRFP-CDH1 was reduced compared to WT. (**F**) The alignment of the SHLD3 RFIP motif to the proposed CDH1 RFIP motif, revealing conserved residues shared in both proteins. (**G**) HA-CDH1 F48A exhibited binding to MAD2L2. HEK293 cells were co-transfected with the indicated plasmids, and α-GFP IP was performed to assess MAD2L2-CDH1 binding. The binding of both HA-CDH1 WT and F48A was detected. (**H**) HA-CDH1 F48A binding to endogenous CDC27 (APC3) was reduced. HEK293 cells were co-transfected with HA CDH1 WT or F48A mutant, and α-HA IP was performed to assess CDC27 CDH1 binding.

To investigate the significance of MAD2L2 dimerization in its role as a regulator of APC/C, we introduced the established dimer-disrupting (DD) [12] mutations at the dimerization interface of MAD2L2. We generated the following mutants with HA or myc tags, namely MAD2L2^R124^, MAD2L2^K44A,R124A^ (2DD), and MAD2L2^K44A,R124A,A135D^ (3DD), as represented in Figure 1A. To confirm the impact of these mutations on MAD2L2 homodimerization, a series of co-immunoprecipitation (co-IP) experiments were conducted. We co-transfected HEK-293 cells with YFP-MAD2L2 along with myc-MAD2L2 WT or myc-DD mutants and subsequently performed α-myc immunoprecipitation (IP). Indeed, both myc-2DD and myc-3DD mutants exhibited reduced binding to YFP-MAD2L2 in comparison to myc-MAD2L2 WT. This suggests that these mutations impede the homodimerization of wild-type MAD2L2 with the mutated MAD2L2 species (Figure 1B). Intriguingly, we often observed that MAD2L2^R124A^ expression was low and seemed to affect the expression levels of other proteins (Supplementary Figure S1J). Notably, the expression level of YFP-MAD2L2 or myc-CDH1 was also reduced to undetectable levels (Figure 1B and Figure 1C, upper panels, respectively). Thus, despite the well-documented literature on the disruptive effect of the R124A mutation on the homodimerization interface, we were unable to validate its dimer-disrupting activity in vivo [11,24]. Next, we aimed to investigate whether the binding of CDH1 to MAD2L2 requires MAD2L2 to be in its homodimeric form. To address this, we co-transfected HEK-293 cells with myc-CDH1 and HA-MAD2L2 WT or HA-dimerization mutants and performed an α-myc IP. We observed that CDH1 binds well to MAD2L2 2DD, whereas the binding of CDH1 to MAD2L2 3DD was diminished by approximately 70% (Figure 1C,D). Considering that the only dissimilarity between 2DD and 3DD lies in the A135D point mutation, we hypothesize that CDH1 binding to MAD2L2 is also influenced by residue A135 rather than solely relying on the C-terminal residue L186 [35]. Moreover, as shown in Figure 1B, the 2DD MAD2L2 mutant disrupts dimer formation. Therefore, the observation that CDH1 can readily bind to MAD2L2 2DD suggests that CDH1 may not require the homodimerization of MAD2L2 for its binding.

To explore the unexpected involvement of MAD2L2’s A135 residue for CDH1 binding, we co-transfected HEK-293 cells with mRFP-CDH1, together with HA-MAD2L2 WT or HA-MAD2L2 A135D mutant, and performed an α−HA IP. Here, we observed that the A135D mutant showed about 40% reduction in CDH1 binding compared to WT MAD2L2, indicating that this A135 residue plays a role in CDH1-MAD2L2 complex formation (Figure 1E). Similarly, when we co-transfected HEK-293 cells with YFP-CDH1 or myc-CDH1 and HA-MAD2L2 WT or HA-MAD2L2 A135D mutant and repeated the α-HA IP, we observed the same reduction (Figure S1E, middle and right panel, respectively). Based on these results, we propose that MAD2L2’s residue A135 might serve as an additional CDH1 binding site, working in conjunction with the established binding site at L186. Residue A135 was shown to be important not only for MAD2L2’s homodimerization but also for stabilizing MAD2L2-SHLD3 binding [6], particularly through SHLD3’s conserved motif ^37^RFIP^40^, with the specific significance of the phenylalanine F38. Interestingly, we observed that CDH1 possesses an identical motif in its C-BOX, at residues ^47^RFIP^50^ (Figure 1F). To explore this motif within CDH1, we introduced a point mutation to create the HA-CDH1 RAIP (F48A) expression vector. We transfected HEK-293 cells with myc-MAD2L2, along with HA-CDH1 WT or HA-CDH1 F48A mutant, and performed an α−myc IP. As seen in Figure 1G, this mutation seems to have no effect on CDH1-MAD2L2 binding, as both WT and mutated forms of CDH1 were able to bind well to MAD2L2. However, the F48A mutation disrupted the C-BOX, causing a clear reduction, as expected, in CDH1’s ability to bind to the APC/C subunit CDC27 (Figure 1H).

### 2.2. Monomeric MAD2L2 Can Properly Regulate Mitotic Entry

**Figure 2:**
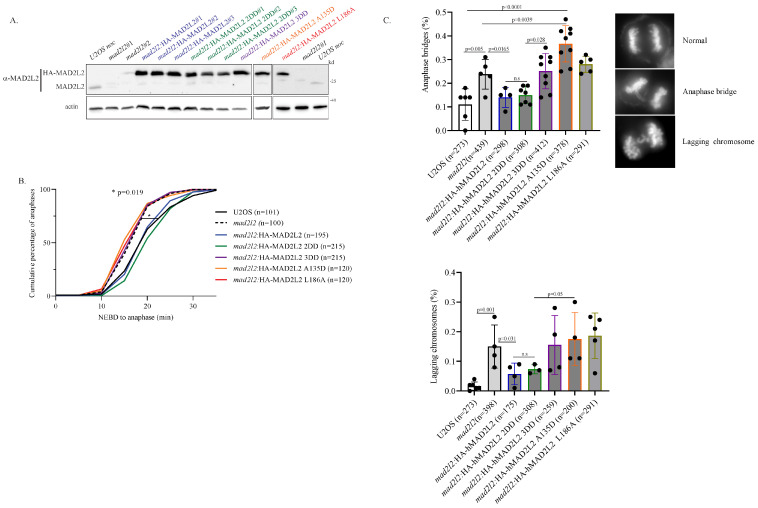
Monomeric MAD2L2 can regulate mitotic entry: (**A**) Clones of U2OS CRISPR/Cas9 knockout *mad2l2* cell line and the stably complemented *mad2l2*#1 cell line with different HA-tagged MAD2L2 mutants, as indicated. (**B**) The quantification of the time taken for knockout U2OS *mad2l2* and the complemented HA-*mad2l2* cells to reach the metaphase–anaphase transition from nuclear envelope breakdown (NEBD), assessed by time-lapse video microscopy (1 frame every 5 min). The plot shows the cumulative percentage of cells that progressed to anaphase. “n” represents the number of cells examined, collected from at least three independent experiments. The *p*-value between U2OS and *mad2l2* cell lines was calculated using a one-tailed *t*-test. (**C**) Percentage of anaphase bridges (upper panel) and lagging chromosomes (lower panel) in *mad2l2* and the complemented HA-*mad2l2* U2OS cell lines. “n” represents the number of anaphases examined; error bar = 1 SD; n.s-not significant; *p*-value between the indicated cell lines was calculated using a one-tailed *t*-test.

Although MAD2L2 homodimerization was found to be essential for the proper activity of both TLS and shieldin, limited information is available regarding MAD2L2’s dimerization state both in mitosis and as part of the MAD2L2-CDH1 complex. Therefore, we were interested in whether MAD2L2’s homodimerization plays a role in mitotic regulation. Previous studies on MAD2L2’s mitotic role have primarily relied on siRNA silencing experiments [34,37]. However, in order to determine the specific effects of different MAD2L2 mutants, we generated a U2OS CRISPR/Cas9 knockout *mad2l2* cell line, where MAD2L2 expression was silenced. This cell line was stably complemented with different HA-tagged MAD2L2 mutants as follows (Figure 2A and Figure S2A–D): MAD2L2 WT (*mad2l2*:HA-MAD2L2); MAD2L2 dimer-disrupting double-mutation K44A + R124A (*mad2l2*:HA-MAD2L2 2DD); MAD2L2 dimer-disrupting triple-mutation K44A + R124A + A135D (*mad2l2*:HA-MAD2L2 3DD); and CDH1-reduced binding mutants A135D and L186A (*mad2l2*:HA-MAD2L2 A135D and *mad2l2*:HA-MAD2L2 L186A). The MAD2L2 L186A serves as a control, as it presents reduced CDH1 binding and is still able to support the formation of the MAD2L2 homodimer [35]. In cases where multiple stable clones were obtained, a single clone was selected, after confirming similar mitotic characteristics.

Since MAD2L2 regulates mitotic progression by binding to CDH1 and preventing it from prematurely activating the APC/C, we examined the potential influence of these dimer-disrupting mutants of MAD2L2 on normal mitotic progression. Using time-lapse microscopy, we measured, for each cell line, the time taken from nuclear envelope breakdown (NEBD) to the metaphase–anaphase transition. As anticipated, *mad2l2* cells exhibited a significantly faster metaphase–anaphase transition compared to WT cells (Figure 2B, represented by the dotted and solid black lines, respectively). This accelerated transition is likely attributed to uncontrolled APC/C activation by free CDH1, resulting in premature anaphase onset. Notably, this phenotype was rescued by both *mad2l2*:HA-MAD2L2 WT and *mad2l2*:HA-MAD2L2 2DD cells (Figure 2B, blue and green lines, respectively). These findings support the notion that the dimer-disrupting 2DD mutation does not prevent MAD2L2 from binding to CDH1 and functions as an effective mitotic regulator. Consequently, these results imply that MAD2L2 homodimerization is not essential for regulating entry into anaphase. Moreover, *mad2l2*:HA-MAD2L2 3DD, *mad2l2*:HA-MAD2L2 A135D, and *mad2l2*:HA-MAD2L2 L186A cell lines failed to rescue this phenotype and exhibited rapid progression to anaphase, similar to the *mad2l2* cells (Figure 2B, indicated by the purple, orange, red, and dotted lines, respectively). As these three cell lines lack proper binding to CDH1, these data agree with the canonical model where the MAD2L2-CDH1 complex is central for mitotic regulation. Moreover, these findings suggest that MAD2L2 homodimerization is dispensable for mitotic entry since *mad2l2*:HA-MAD2L2 2DD, which retains its ability to bind CDH1 but does not form a dimer, effectively rescues the WT phenotype. Unregulated rapid entry into mitosis typically leads to compromised mitotic fidelity. Indeed, MAD2L2 silencing was previously reported to induce mitotic aberrations such as anaphase bridges and lagging chromosomes [34,37]. To investigate the importance of MAD2L2 dimerization for mitotic fidelity, we monitored the percentage of anaphase bridges (Figure 2C, upper panel) and lagging chromosomes (Figure 2C, lower panel) in all the complemented cell lines. Consistent with previous studies, *mad2l2* cells presented high levels of these mitotic aberrations compared to WT cells. Once again, this phenotype was rescued by both HA-MAD2L2 WT and HA-MAD2L2 2DD complemented cells, resulting in a reduction in their occurrence, with HA-MAD2L2 2DD displaying a more effective rescue phenotype (Figure 2C, blue and green bars, respectively). Both HA-MAD2L2 3DD and HA-MAD2L2 A135D complemented cells failed to decrease the frequency of anaphase bridges, with A135D complemented cells presenting an even worse phenotype than *mad2l2* cells (Figure 2C, purple and orange bars, respectively). This further supports the notion that the MAD2L2-CDH1 complex formation, but not MAD2L2 homodimerization, is essential in maintaining mitotic fidelity.

**Figure 3:**
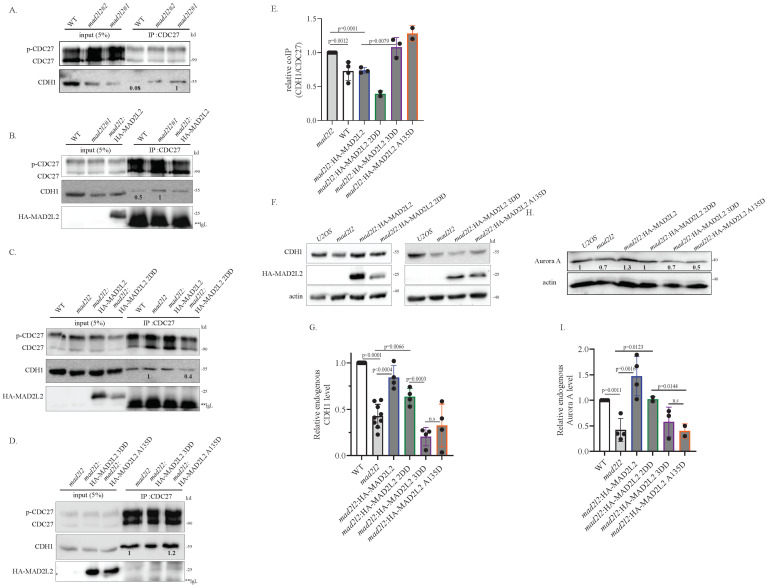
Monomeric MAD2L2 inhibits APC/C activation: (**A**) *mad2l2* clones present premature binding of CDH1 to the APC/C. In order to assess the binding of CDH1 to the APC/C in each cell line, an α-CDC27 IP was performed on U2OS and *mad2l2* clones that were arrested in nocodazole for 16h. In both *mad2l2* clones, but not in WT cells, CDH1 prematurely binds to the CDC27 subunit of the APC/C, during nocodazol arrest. (**B**) *mad2l2*:HA-MAD2L2 restores the canonical binding pattern of CDH1 to the APC/C and prevents premature binding of CDH1 to the APC/C. In order to assess the binding of CDH1 to the APC/C in each cell line, an α-CDC27 IP was performed on cells arrested in nocodazole for 16h. WT and *mad2l2*:HA-MAD2L2 cells present lower binding to the CDC27 subunit, compared to *mad2l2* cells. (**C**) *mad2l2*:HA-MAD2L2 2DD is able to prevent the premature binding of CDH1 to the APC/C. In order to assess the binding of CDH1 to the APC/C in each cell line, an α-CDC27 IP was performed on cells arrested in nocodazole for 16 h. WT and *mad2l2*:HA-MAD2L2 2DD present lower binding present lower binding to the CDC27 subunit, compared to *mad2l2* cells. (**D**) Both *mad2l2*:HA-MAD2L2 3DD and *mad2l2*:HA-MAD2L2 A135D present premature binding of CDH1 to the APC/C and fail to restore the normal binding pattern, as indicated by the relatively high level of CDH1 bound to CDC27 during nocodazole treatment. **IgL–IgG Light chain. (**E**) The quantification of the relative amount of CDH1 bound to CDC27 in all presented cell lines. Each dot represents an independent repeat of the IP. The blots contributing to this analysis are shown in Figure S3A–E. (**F**) Premature binding of CDH1 to the APC/C, during nocodazole arrest, leads to a reduction in CDH1 levels. Endogenous levels of CDH1 were monitored in the indicated cell lines after 16 h of nocodazole treatment. Lack of MAD2L2, or the presence of MAD2L2 without the ability to bind CDH1, leads to premature CDH1 degradation. (**G**) The quantification of the relative amount of endogenous CDH1 in nocodazole-arrested cells. Each dot represents an independent repeat. Blots contributing to this analysis are shown in Figure S3F. (**H**) Premature binding of CDH1 to the APC/C, during nocodazole arrest, leads to a reduction in Aurora A levels. Endogenous levels of Aurora A were monitored in the indicated cell lines after 16h of nocodazole treatment. Lack of MAD2L2, or the presence of MAD2L2 without the ability to bind CDH1, leads to premature Aurora A degradation. (**I**) The quantification of the relative amount of endogenous Aurora A in nocodazole-arrested cells. Each dot represents an independent repeat. The additional blots contributing to this analysis are shown in Figure S3I. For all: Error bar = 1 SD; *p*-value was calculated using a one-tailed *t*-test.

Next, we explored APC/C activation during nocodazole arrest. Normally, nocodazole treatment synchronizes cells at prometaphase, where the cells arrest with an active spindle assembly checkpoint (SAC). During this arrest, the APC/C is inactive and CDH1 does not bind to the APC/C. We aimed to assess premature APC/C^CDH1^ activity by comparing the relative levels of CDH1 that bind to the APC/C prematurely, during nocodazole arrest. Following prometaphase synchronization, we performed an IP of the APC/C subunit CDC27 and monitored the amount of CDH1 bound to it. Our results revealed that the amount of CDH1 bound to CDC27 in nocodazole-arrested *mad2l2* cells was significantly higher compared to U2OS WT (Figure 3A), indicating that the absence of MAD2L2 allows for the premature binding of CDH1 to the APC/C. This phenotype was rescued by complementation with *mad2l2*:HA-MAD2L2 WT, as seen by a reduction in the levels of APC/C-bound CDH1 toward the WT levels (Figure 3B). Furthermore, the *mad2l2*:HA-MAD2L2 2DD cells also exhibited rescue, with lower co-IP’d CDH1 levels compared to the *mad2l2* cells (Figure 3C). In contrast, both *mad2l2*:HA-MAD2L2 3DD and *mad2l2*:HA-MAD2L2 A135D complemented cells maintained the unregulated APC/C phenotype observed in the *mad2l2* cells, as indicated by the relatively high level of CDH1 bound to CDC27 (Figure 3D). To quantify the relative amount of bound CDH1, we normalized all results to the amount of CDC27-bound CDH1 co-IP’d from *mad2l2* cells (Figure 3E). Based on these findings, we can conclude that the 3DD and A135D MAD2L2 mutants, which do not bind well to CDH1, are also unable to regulate APC/C activation, while MAD2L2 homodimerization ability has no impact on APC/C^CDH1^ formation. These results agree with our previous data, suggesting that homodimerization of MAD2L2 appears to be dispensable for its mitotic functions.

Next, we investigated whether the observed premature binding of CDH1 to the APC/C has a biological relevance and can indeed lead to unscheduled APC/C activation. To evaluate unscheduled APC/C activation, we used nocodazole-arrested cells, where APC/C substrates accumulate at relatively high levels [34,38]. Unscheduled APC/C activation can be detected by premature substrate degradation; hence, we decided to monitor the endogenous levels of three APC/C^CDH1^ substrates, CDH1 [32], Aurora A [39], and Cyclin B1 [40], in nocodazole arrested cells as an indicator of the relative APC/C activity. After 16 h of nocodazole treatment, the endogenous protein levels were compared between *mad2l2* and complemented cell lines. As anticipated, we observed a reduction in all examined substrates in the *mad2l2* cells compared to WT cells, reflecting unregulated APC/C^CDH1^ activation likely due to the absence of MAD2L2 (Figure 3F–I and Figure S3F–J). The WT phenotype was rescued in the *mad2l2*:HA-MAD2L2 WT and in the *mad2l2*:HA-MAD2L2 2DD complemented cells, supporting our previous finding that MAD2L2 can regulate mitotic progression in its monomeric form. However, the non-CDH1-binding mutants *mad2l2*:HA-MAD2L2 3DD and *mad2l2*:HA-MAD2L2 A135D failed to rescue the premature degradation, presenting reduced levels similar to those of *mad2l2* cells. The lower substrate levels in the 3DD and A135D cell lines indicate that the APC/C is activated in an uncontrolled manner, leading to earlier substrate degradation. To quantify the relative amount of CDH1, Aurora A, and Cyclin B1 (Figure S3J), we normalized all results to the protein levels in WT cells. These data further emphasize the importance of the MAD2L2-CDH1 complex in mitotic regulation and for the timely degradation of APC/C substrates and keeping mitotic fidelity. Moreover, this reinforces the observation that MAD2L2 can function as a mitotic regulator in its monomeric form.

### 2.3. CDH1 Overexpression Reduces MAD2L2 Homodimerization

**Figure 4:**
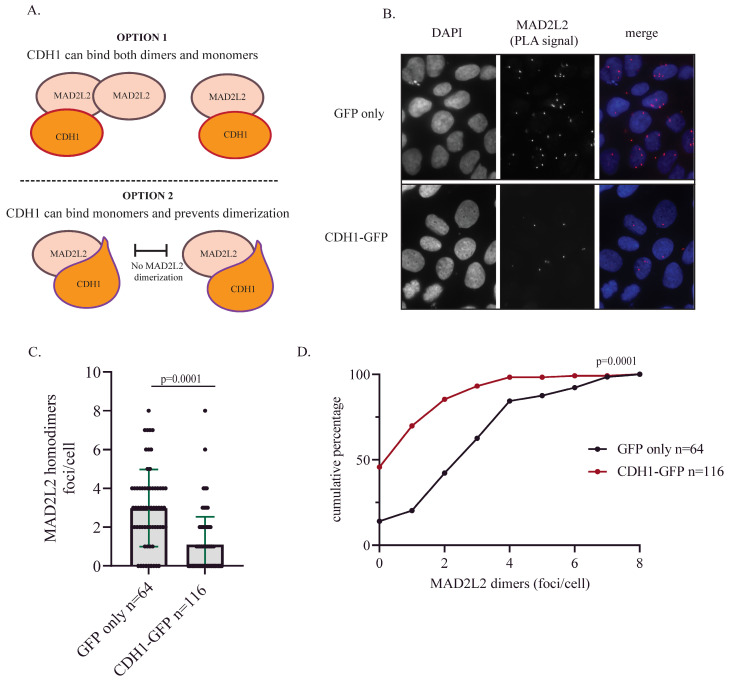
CDH1 overexpression reduces MAD2L2 homodimerization: (**A**) A graphic model presenting two options for MAD2L2-CDH1 interaction. Option 1: CDH1 can bind both monomeric and dimeric forms of MAD2L2 via the L186 residue. Option 2: CDH1 binds to the monomeric form of MAD2L2 using both L186 and A135 residues, blocking MAD2L2 homodimerization. (**B**) In situ MAD2L2 homodimer formation is reduced upon CDH1-GFP overexpression, as indicated by a reduced number of foci/cell. Representative images of the PLA foci. (**C**) The quantification of MAD2L2 homodimer from the PLA assay; error bar = 1 SD; each dot represents a cell. (**D**) The number of foci/cell for each cell line in a cumulative percentage plot to observe the difference between cell populations upon treatments; *p*-value was calculated using a one-tailed *t*-test.

The finding that MAD2L2 can function as a monomer during mitosis provides insight into the mechanism that regulates decision-making in MAD2L2’s complex formation. Given that CDH1 levels start to accumulate from the early stages of the S phase, it is crucial for MAD2L2 to bind to CDH1 in a stable manner until the onset of anaphase. Whether CDH1 can interact with either MAD2L2 monomers or dimers (Figure 4A, option 1) and whether CDH1 binding prevents MAD2L2 homodimerization (Figure 4A, option 2) are still open questions. To test whether the binding of CDH1 can potentially reduce MAD2L2 homodimerization, we transfected U2OS WT cells with either a GFP vector or GFP-CDH1 and assessed the MAD2L2 homodimerization using the proximity ligation assay (PLA). Our data suggest that CDH1 overexpression leads to a decrease in MAD2L2 homodimer formation, indicating a clear preference for monomeric binding of MAD2L2 to CDH1 (As in Figure 4A, option 2). Cells transfected with the GFP vector presented a relatively higher number of foci per cell, with three to eight foci detected in over 50% of cells. In contrast, cells transfected with GFP-CDH1 showed a significant reduction in the number of foci per cell (Figure 4B,C). In the CDH1-GFP transfected cells, we observed an increase from 15% to almost 50% in the number of cells with no foci at all, and the remaining cells had fewer than four foci (Figure 4D). These data suggest that once MAD2L2 is bound to CDH1, it becomes unavailable to participate in other complexes and remains in its monomeric configuring.

## 3. Discussion

Despite MAD2L2 being a central regulatory protein, the role of MAD2L2 homodimerization in mitosis has remained less studied. Here, we asked whether MAD2L2 homodimerization has a role in both mitotic regulation and the formation of the MAD2L2-CDH1 complex. Our findings demonstrate that monomeric MAD2L2 fully retains its function as a mitotic regulator, differently than in DNA damage response pathways, which require MAD2L2 homodimerization for proper complex formation and function [6,12] 

During mitosis, MAD2L2 interacts with CDH1 to prevent it from prematurely activating the APC/C. This interaction is essential for maintaining accurate mitotic progression and fidelity. We used a dimer-disrupting mutant of MAD2L2, named 2DD (K44A + R124A), to demonstrate that CDH1 can bind MAD2L2 in its monomeric state. However, our results showed that the 3DD mutant (K44A + R124A + A135D), which carries an additional point mutation, namely A135D, failed to interact with CDH1 (Figure 1B–E). We, therefore, concluded that CDH1’s failure to interact with MAD2L2 3DD was not due to its forced monomeric state but rather due to the presence of the added A135D mutation. We speculated that A135 may serve as a novel direct binding residue for CDH1, and indeed, we confirmed that a single A135D mutation in MAD2L2 reduced the binding of CDH1, emphasizing the significance of this specific residue in the interaction between MAD2L2 and CDH1.

This unexpected finding adds a new layer of complexity to our understanding of MAD2L2’s interactions. We classified CDH1 as a Rev1-like binding partner of MAD2L2, based on its binding to the C-terminus of MAD2L2 involving the residue L186 [35]. This interaction pattern is similar to Rev1’s interaction with the far C-terminus of MAD2L2, through an established binding interface involving L186, Q200, and Y202 of MAD2L2 [11]. Although A135 has not been shown to be involved with Rev1 binding, it is important in MAD2L2’s interaction with SHLD3. In the shieldin complex, A135 interacts with the phenylalanine residue of the SHLD3 at the ^37^RFIP^40^ motif via hydrophobic interactions. Interestingly, we found that CDH1 carries a similar motif in its C-box ^47^RFIP^50^; however, the corresponding phenylalanine in CDH1-F48A did not play a role in MAD2L2 binding, as the F48A mutant CDH1 still bound MAD2L2 normally or even stronger. This discrepancy may arise from the fact that SHLD3 interacts with homodimeric MAD2L2, and the larger phenylalanine residue is required to bridge MAD2L2 dimer and SHLD3 interaction, where the substitution of phenylalanine to the smaller alanine destabilizes the interaction. In contrast, as suggested here, CDH1 interacts with monomeric MAD2L2, and the smaller alanine residue in the CDH1 F48A mutant may still be sufficient for the binding at residue A135 of MAD2L2, as it does not require dimer bridging. Moreover, both phenylalanine and alanine are hydrophobic residues, suggesting that they can interact similarly with the hydrophobic A135 residue on MAD2L2. Notably, the A135D mutation in MAD2L2 reduced the binding to CDH1, as the hydrophobic A135 was substituted with the hydrophilic aspartic acid. This mutation may disrupt a hydrophobic interaction interface with the F48 residue of CDH1 (Figure 1G). So, whether MAD2L2 A135 directly interacts with CDH1 F48 residue still remains an open question in our effort to map the CDH1-MAD2L2 complex.

The generation of a stable MAD2L2-CDH1 complex is important for proper mitosis. Our data suggest that monomeric MAD2L2 binds CDH1 and that this is sufficient for the regulation of mitotic progression. The lack of MAD2L2 causes the deregulation of mitosis, as observed in our *mad2l2* knockout cells presenting a rapid onset of anaphase. Specifically, cells stably complemented with a non-CDH1-binding MAD2L2 species, such as the L186A, 3DD, or A135D, exhibited a fast anaphase onset similar to the knockout cells. However, the 2DD monomeric MAD2L2 displayed a normal rate of mitotic progression. Moreover, the non-CDH1 binding mutants failed to maintain mitotic fidelity, presenting an elevated incidence of anaphase bridges and lagging chromosomes, whereas the monomeric 2DD MAD2L2 rescued these mitotic aberrations (Figure 2). Interestingly, we observed that the A135D complemented cell line consistently presented the worse phenotype regarding mitotic aberrations, and their growth was relatively faster compared to the other cell lines. A135 is a central residue for general MAD2L2 activity including homodimerization, and interaction with CDH1 and maybe other less characterized MAD2L2 binding proteins. Conceivably, A135D might form a weak dimer, through which it can still bind other proteins but lacks full activity, gaining a dominant-negative (DN) feature, thus preventing the proper activity of the different MAD2L2-containing complexes. As MAD2L2 dimerization is mainly important for DNA repair and TLS, its influence is more pronounced in the appearance of anaphase bridges. Mutating this residue alone seems more harmful than mutating the three central residues involved in dimerization (3DD mutant). This might support the DN hypothesis as the 3DD cannot participate in the MAD2L2 complexes, and the cells eventually adapt to the lack of MAD2L2, similar to *mad2l2* knockout.

Furthermore, we confirmed that the accelerated onset of anaphase observed in cells lacking functionally active MAD2L2 was due to premature CDH1 binding to the APC/C (Figure 3). We demonstrated that the monomeric 2DD MAD2L2 could prevent premature CDH1 binding to the APC/C. Moreover, the monomeric 2DD form of MAD2L2 was able to restore normal endogenous levels of APC/C substrates such as Cyclin B1, CDH1, and Aurora A kinase in prometaphase-synchronized cells, indicating it can properly inhibit the APC/C during nocodazole block. Our findings confirm that monomeric MAD2L2 can bind to CDH1 and maintain functionality as it prevents premature APC/C activation and prevents the appearance of mitotic aberrations.

Recent work by Vassel et al. [36] confirmed and reinforced the importance of MAD2L2 dimerization for survival during different types of DNA damage and for TLS, shieldin, and fork progression. In addition, their findings suggest that MAD2L2 dimerization is almost dispensable for chromosome segregation and cell cycle regulation. In agreement with their findings, we present several aspects of mitotic regulation that are not dependent on MAD2L2 dimerization. One of the main functions of MAD2L2 in early mitosis is limiting the access of CDH1 to the APC/C, protecting the cell from premature APC/C activation.

Lastly, the finding of A135 as an additional CDH1 binding site in MAD2L2 suggests a possible mechanism for the preferential formation of specific MAD2L2 complexes. Selective binding interaction was observed in shieldin, where SHLD3 blocked the binding between Rev1 and MAD2L2, thus inhibiting MAD2L2’s TLS function and directing it toward its role in non-homologous end joining (NHEJ). Thus, a potentially comparable mechanism with CDH1-MAD2L2 binding is possible, where CDH1 binds a monomeric MAD2L2 through interactions with both A135 at the dimerization interface and L186 at the C-terminus (Figure 4). This binding may prevent MAD2L2 homodimerization, inhibiting its function in TLS and shieldin, which require MAD2L2 homodimerization for proper complex function. The PLA data (Figure 4) support this model as the formation of MAD2L2 homodimers was reduced after CDH1 overexpression, suggesting that the dimerization state of MAD2L2 may be another mechanism of differentiation between its functions, where CDH1 binding keeps MAD2L2 in its monomeric state, excluding it from DNA damage response roles and guarding the necessary pool of MAD2L2 needed for mitosis regulation.

## 4. Materials and Methods

### 4.1. Expression Vectors and Site-Directed Mutagenesis

The MAD2L2 and CDH1-tagged versions are all in the pCDNA3.1 plasmid. Mutants were prepared by PCR amplification. Point mutations were introduced using a PCR-based method. The PFU Turbo DNA polymerase (600250-52) was used according to Agilent’s QuikChange site-directed mutagenesis protocol.

### 4.2. Cell Culture, Transfection, and Synchronization

HEK293 and U2OS cells were cultured in DMEM (BI; 01-052-1A) with 4.5 g/L D-glucose, 4 mM L-glutamine, 10% fetal bovine serum (BI; 04-007-1A), and 1% penicillin/streptomycin. Transfections were performed using the Avalanche^®^ Everyday Transfection Reagent (EZT-EVDY-1) according to the manufacturer’s protocol. U2OS cell synchronization in prometaphase was performed by treatment with 0.2 µM nocodazole (A8487-10; A2S Technologies). Cells were treated for 14–16 h and then harvested by gentle shake-off for subsequent protein extraction.

### 4.3. CRISPR/Cas9 Knockdown

Guide RNA sequences targeting MAD2L2 were phosphorylated, annealed, and ligated into a pX330-U6-Chimeric_BB-CBh-hSpCas9 vector (#42230, Addgene, a gift from Dr. Gabi Gerlitz Ariel University). The resulting plasmid was validated via sequencing and transfected into U2OS cells, along with pGK-puro puromycin resistance plasmid (gift from Gabi Gerlitz, #11349 Addgene) at a 9:1 ratio, respectively, using the Avalanche^®^ Everyday Transfection Reagent (EZT-EVDY-1, College Park, Maryland, USA) according to the manufacturer’s protocol. Then, 24 h following transfection, puromycin was added at a 2 µg/mL concentration to select for transfected cells. Cells were maintained in media containing 2 µg/mL puromycin until the formation of isolated colonies. Single clones were harvested after 30 days, and MAD2L2 knockout was validated using Western blot. Two successful knockout clones of *mad2l2* U2OS cells were isolated, and one clone was selected for use as the basis for subsequent MAD2L2 complementation.

### 4.4. Stable Complementation

HA-tagged MAD2L2 plasmids (WT or mutant species) were transfected to *mad2l2* U2OS cells, along with the pGK-hygro hygromycin-resistance plasmid (a gift from Dr. Gabi Gerlitz) at a 9:1 ratio, respectively. Then, 24 h following transfection, hygromycin was added at a 100 µg/mL concentration to select for transfected cells. Cells were maintained in media containing 100 µg/mL hygromycin until the formation of isolated colonies. Single clones were harvested after 30 days, and MAD2L2 complementation for each stable species was validated using Western blot.

### 4.5. Western Blot, Immunoprecipitation, and Antibodies

For immunoblotting and IP, cells were lysed in extraction buffer containing 50 mM Tris-HCl pH 8, 150 mM NaCl, 20 mM EGTA, 50 mM NaF, and 1% Triton X-100, supplemented with protease inhibitor (Merck 539134, Arklow, Ireland). Cells were lysed on ice for 30 min and cleared by centrifugation at 20,000× *g* for 30 min at 4 °C. For immunoblotting, extracts were boiled in Laemmli buffer for 5 min. Equal amounts of protein sample (30 µg) were loaded on 10% acrylamide gels and transferred to a nitrocellulose membrane (Amershem, GE10600002, Amershem, UK). For IP, the clarified lysates were supplemented with the appropriate primary antibody and incubated for 1 h at 4 °C. Next, 30 µL of equilibrated protein G–Sepharose beads (Merck-Millipore 16-266, Arklow, Ireland) was added for 30 min. Finally, beads were washed twice in PBS buffer and boiled in Laemmli buffer for 5 min. The primary antibodies used at 1:1000 dilution were: CDC27, (BD Biosciences, 610454, Franklin Lakes, NJ, USA), MAD2L2 mouse (BD Biosciences, 612266), CDH1 mouse (Merck, CC-43, Arklow, Ireland), Aurora A (BD Biosciences, 610936, NJ, USA) Cyclin B1 (Santa Cruz sc-245, Dallas, TX, USA), Myc (Santa Cruz; sc-40, Dallas, TX, USA) and HA (Santa Cruz; sc-7392, Dallas, TX, USA). Actin (Abcam, ab8227-50, Cambridge, UK) was used at 1:5000 dilution. HRP IgG light chain-specific anti-mouse secondary antibody (115-035-174; Jackson ImmunoResearch Laboratories, Inc., Ely CB7 4EX, UK.) or HRP IgG light chain-specific anti-rabbit secondary antibody (211-032-171; Jackson ImmunoResearch Laboratories, Inc., Ely CB7 4EX, UK.) was used at 1:10,000 dilution.

### 4.6. Time-Lapse Imaging and Analysis

Time-lapse imaging was performed on an Olympus IX81 (Tokyo, Japan), fitted with a modified incubation chamber to maintain the cells at 37 °C and 7% CO_2_. Images were acquired every 5 min using a 20X objective for 15–18 h. The time from nuclear envelope breakdown to anaphase was measured for individual cells in the stable cell lines, and the cumulative percentage distribution was calculated.

### 4.7. Chromosome Spreads

U2OS cells were grown on glass coverslips in 6-well plates and fixed in 4% paraformaldehyde for 10 min at room temperature. Nuclei were stained with DAPI (1:1000 dilution) at room temperature in the dark for 3 min. Coverslips were mounted on glass slides and imaged using the Olympus IX81 microscope. The fraction of mitotic cells exhibiting chromosomal aberrations (anaphase bridges) was calculated for each stable cell line.

### 4.8. Proximity Ligation Assay

Protein–protein interactions were measured using the NaveniFlex^TM^ (NF.MR.100) proximity ligation assay platform, according to the manufacturer’s instructions. Briefly, the selected cells were grown in black 96-well plates with clear flat well bottoms and fixed in 4% paraformaldehyde for 10 min at room temperature. Next, cells were permeabilized in 0.5% TritonX100 in PBSx1 for 10 min at room temperature and then blocked in the NaveniFlex blocking buffer for 1 h at 37 °C. The primary monoclonal antibodies used in the proximity ligation assay included MAD2L2 rabbit (ABClonal, A4630, Woburn, MA, USA) and CDH1 mouse (Merck, CC-43, Arklow, Ireland). The selected primary antibody pair (mouse/rabbit) was diluted 1:200 in the NaveniFlex diluent and incubated for 1 h at 37 °C. Proprietary secondary Navenibodies were applied for 1 h at 37 °C. The Navenibody oligo activation, ligation, and amplification steps were performed at 37 °C with the relevant buffers and enzyme mixes. The coverslips were washed between each step with 0.1% PBSx1-Tween. Nuclei were stained with DAPI (1:2000 dilution) at room temperature in the dark for 5 min. Cells were imaged using the Olympus IX81. In each treatment, we used α-rabbit MAD2L2 and α-mouse CDH1 to investigate the formation of the PLA complex and quantify the number of foci per cell, representing the number of MAD2L2 homodimers formed.

## Data Availability

The authors confirm that the data supporting the findings of this study are available within the article and its Supplementary Materials. Additional information will be willingly shared upon request.

## Supplementary Materials

The following supporting information can be downloaded at: https://www.mdpi.com/article/10.3390/ijms252111485/s1.

## References

[1] Chatterjee N., Walker G.C. (2017). Mechanisms of DNA damage, repair, and mutagenesis. Environ. Mol. Mutagen..

[2] Tubbs A., Nussenzweig A. (2017). Endogenous DNA Damage as a Source of Genomic Instability in Cancer. Cell.

[3] Muniyappa K., Kshirsagar R., Ghodke I. (2014). The HORMA domain: An evolutionarily conserved domain discovered in chromatin-associated proteins, has unanticipated diverse functions. Gene.

[4] de Krijger I., Boersma V., Jacobs J.J. (2021). REV7: Jack of many trades. Trends Cell Biol..

[5] Wang X., Pernicone N., Pertz L., Hua D., Zhang T., Listovsky T., Xie W. (2019). REV7 has a dynamic adaptor region to accommodate small GTPase RAN/Shigella IpaB ligands, and its activity is regulated by the RanGTP/GDP switch. J. Biol. Chem..

[6] Liang L., Feng J., Zuo P., Yang J., Lu Y., Yin Y. (2020). Molecular basis for assembly of the shieldin complex and its implications for NHEJ. Nat. Commun..

[7] Sale J.E., Lehmann A.R., Woodgate R. (2012). Y-family DNA polymerases and their role in tolerance of cellular DNA damage. Nat. Rev. Mol. Cell Biol..

[8] Vaisman A., Woodgate R. (2017). Translesion DNA polymerases in eukaryotes: What makes them tick?. Crit. Rev. Biochem. Mol. Biol..

[9] Livneh Z., Z O., Shachar S. (2010). Multiple two-polymerase mechanisms in mammalian translesion DNA synthesis. Cell Cycle.

[10] Lee Y.-S., Gregory M.T., Yang W. (2014). Human Pol ζ purified with accessory subunits is active in translesion DNA synthesis and complements Pol η in cisplatin bypass. Proc. Natl. Acad. Sci..

[11] Hara K., Hashimoto H., Murakumo Y., Kobayashi S., Kogame T., Unzai S., Akashi S., Takeda S., Shimizu T., Sato M. (2010). Crystal Structure of Human REV7 in Complex with a Human REV3 Fragment and Structural Implication of the Interaction between DNA Polymerase ζ and REV1. J. Biol. Chem..

[12] Rizzo A.A., Vassel F.-M., Chatterjee N., D’souza S., Li Y., Hao B., Hemann M.T., Walker G.C., Korzhnev D.M. (2018). Rev7 dimerization is important for assembly and function of the Rev1/Polζ translesion synthesis complex. Proc. Natl. Acad. Sci. USA.

[13] Rizzo A.A., Korzhnev D.M. (2019). The Rev1-Polζ translesion synthesis mutasome: Structure, interactions and inhibition. Enzymes.

[14] McPherson K.S., Rizzo A.A., Erlandsen H., Chatterjee N., Walker G.C., Korzhnev D.M. (2022). Evolution of Rev7 interactions in eukaryotic TLS DNA polymerase Polζ. J. Biol. Chem..

[15] Kikuchi S., Hara K., Shimizu T., Sato M., Hashimoto H. (2012). Structural Basis of Recruitment of DNA Polymerase ζ by Interaction between REV1 and REV7 Proteins. J. Biol. Chem..

[16] Pernicone N., Elias M., Onn I., Tobi D., Listovsky T. (2022). Disrupting the MAD2L2-Rev1 Complex Enhances Cell Death upon DNA Damage. Molecules.

[17] Wojtaszek J.L., Chatterjee N., Najeeb J., Ramos A., Lee M., Bian K., Xue J.Y., Fenton B.A., Park H., Li D. (2019). A Small Molecule Targeting Mutagenic Translesion Synthesis Improves Chemotherapy. Cell.

[18] Doles J., Oliver T.G., Cameron E.R., Hsu G., Jacks T., Walker G.C., Hemann M.T. (2010). Suppression of Rev3, the catalytic subunit of Polζ, sensitizes drug-resistant lung tumors to chemotherapy. Proc. Natl. Acad. Sci. USA.

[19] Ghezraoui H., Oliveira C., Becker J.R., Bilham K., Moralli D., Anzilotti C., Fischer R., Deobagkar-Lele M., Sanchiz-Calvo M., Fueyo-Marcos E. (2018). 53BP1 cooperation with the REV7–shieldin complex underpins DNA structure-specific NHEJ. Nature.

[20] Xu G., Chapman J.R., Brandsma I., Yuan J., Mistrik M., Bouwman P., Bartkova J., Gogola E., Warmerdam D., Barazas M. (2015). REV7 counteracts DNA double-strand break resection and affects PARP inhibition. Nature.

[21] Boersma V., Moatti N., Segura-Bayona S., Peuscher M.H., van der Torre J., Wevers B.A., Orthwein A., Durocher D., Jacobs J.J.L. (2015). MAD2L2 controls DNA repair at telomeres and DNA breaks by inhibiting 5′ end resection. Nature.

[22] Noordermeer S.M., Adam S., Setiaputra D., Barazas M., Pettitt S.J., Ling A.K., Olivieri M., Álvarez-Quilón A., Moatti N., Zimmermann M. (2018). The shieldin complex mediates 53BP1-dependent DNA repair. Nature.

[23] Setiaputra D., Durocher D. (2019). Shieldin – the protector of DNA ends. Embo Rep..

[24] de Krijger I., Föhr B., Pérez S.H., Vincendeau E., Serrat J., Thouin A.M., Susvirkar V., Lescale C., Paniagua I., Hoekman L. (2021). MAD2L2 dimerization and TRIP13 control shieldin activity in DNA repair. Nat. Commun..

[25] Clairmont C.S., Sarangi P., Ponnienselvan K., Galli L.D., Csete I., Moreau L., Adelmant G., Chowdhury D., Marto J.A., D’andrea A.D. (2020). TRIP13 regulates DNA repair pathway choice through REV7 conformational change. Nat. Cell Biol..

[26] Chen J., Fang G. (2001). MAD2B is an inhibitor of the anaphase-promoting complex. Genes Dev..

[27] Pfleger C.M., Salic A., Lee E., Kirschner M.W. (2001). Inhibition of Cdh1–APC by the MAD2-related protein MAD2L2: A novel mechanism for regulating Cdh1. Genes Dev..

[28] Sudakin V., Ganoth D., Dahan A., Heller H., Hershko J., Luca F.C., Ruderman J.V., Hershko A. (1995). The cyclosome, a large complex containing cyclin-selective ubiquitin ligase activity, targets cyclins for destruction at the end of mitosis. Mol. Biol. Cell.

[29] King R.W., Peters J.-M., Tugendreich S., Rolfe M., Hieter P., Kirschner M.W. (1995). A 20s complex containing CDC27 and CDC16 catalyzes the mitosis-specific conjugation of ubiquitin to cyclin B. Cell.

[30] Pines J. (2011). Cubism and the cell cycle: The many faces of the APC/C. Nat. Rev. Mol. Cell Biol..

[31] Kramer E.R., Scheuringer N., Podtelejnikov A.V., Mann M., Peters J.-M. (2000). Mitotic Regulation of the APC Activator Proteins CDC20 and CDH1. Mol. Biol. Cell.

[32] Listovsky T., Oren Y.S., Yudkovsky Y., Mahbubani H.M., Weiss A.M., Lebendiker M., Brandeis M. (2004). Mammalian Cdh1/Fzr mediates its own degradation. EMBO J..

[33] Visintin R., Prinz S., Amon A. (1997). *CDC20* and *CDH1*: A Family of Substrate-Specific Activators of APC-Dependent Proteolysis. Science.

[34] Listovsky T., Sale J.E. (2013). Sequestration of CDH1 by MAD2L2 prevents premature APC/C activation prior to anaphase onset. J. Cell Biol..

[35] Pernicone N., Grinshpon S., Listovsky T. (2020). CDH1 binds MAD2L2 in a Rev1-like pattern. Biochem. Biophys. Res. Commun..

[36] Vassel F.M., Laverty D.J., Bian K., Piett C.G., Hemann M.T., Walker G.C., Nagel Z.D. (2023). REV7 Monomer Is Unable to Par-ticipate in Double Strand Break Repair and Translesion Synthesis but Suppresses Mitotic Errors. Int. J. Mol. Sci..

[37] Bhat A., Wu Z., Maher V.M., McCormick J.J., Xiao W. (2015). Rev7/Mad2B plays a critical role in the assembly of a functional mitotic spindle. Cell Cycle.

[38] Brito D.A., Rieder C.L. (2008). The ability to survive mitosis in the presence of microtubule poisons differs significantly between human nontransformed (RPE-1) and cancer (U2OS, HeLa) cells. Cell Motil. Cytoskelet..

[39] Floyd S., Pines J., Lindon C. (2008). APC/CCdh1 Targets Aurora Kinase to Control Reorganization of the Mitotic Spindle at Anaphase. Curr. Biol..

[40] Clute P., Pines J. (1999). Temporal and spatial control of cyclin B1 destruction in metaphase. Nat. Cell Biol..

