# Supplementary material for: MAD2L2 Dimerization Is Not Essential for Mitotic Regulation
Source: Int J Mol Sci. 2024 Oct 25;25(21):11485. doi: 10.3390/ijms252111485 (PMC11545987; doi:10.3390/ijms252111485)
Supplement: Supplementary file 1 [file ijms-25-11485-s001.zip › ijms-3225981-supplementary.pdf]

Figure S1

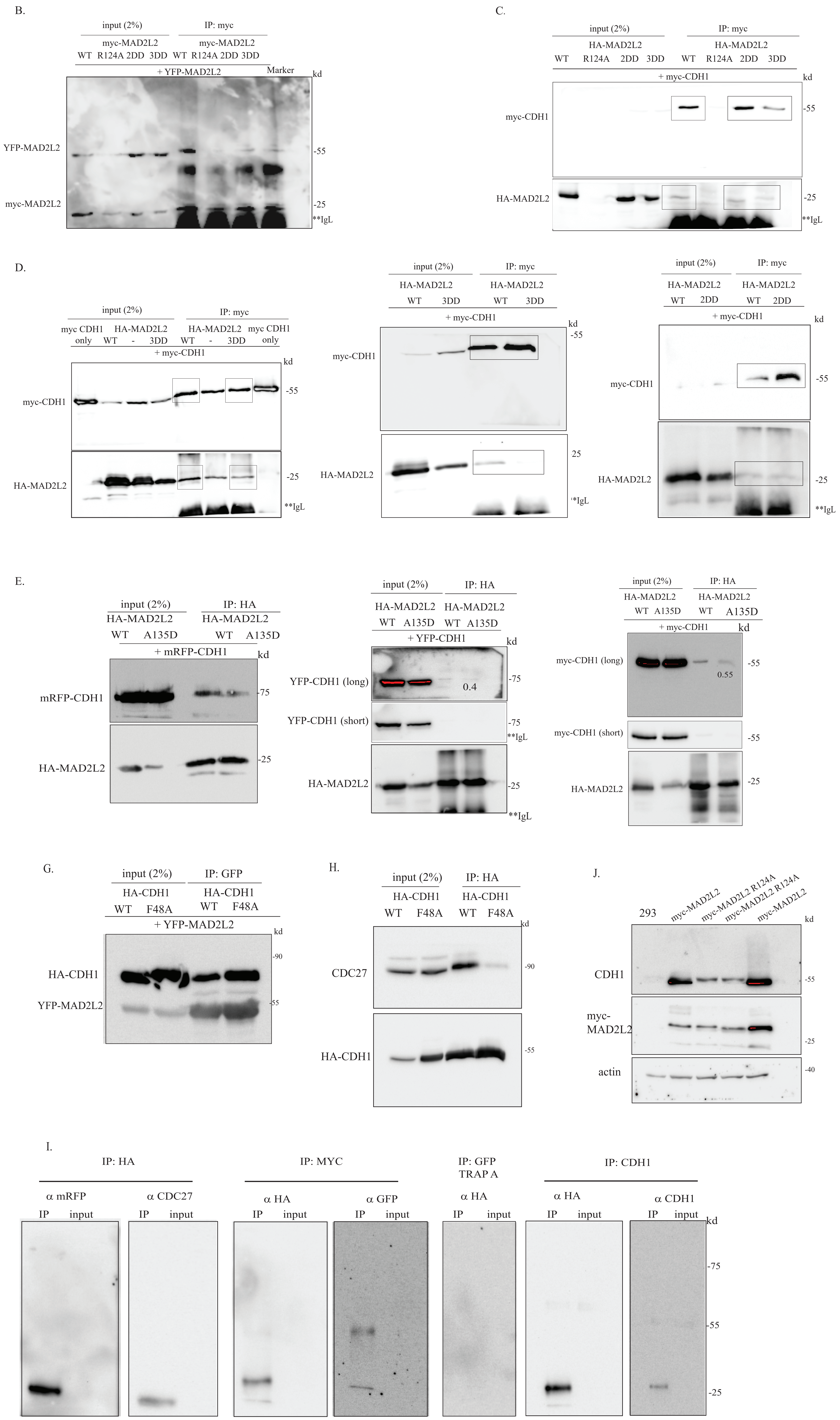
B.
input (2%)
IP: myc
myc-MAD2L2
WT R124A 2DD 3DD WT R124A 2DD 3DD
+ YFP-MAD2L2
Marker
kd
YFP-MAD2L2
myc-MAD2L2
-55
-25
**IgL
C.
input (2%)
IP: myc
HA-MAD2L2
WT R124A 2DD 3DD WT R124A 2DD 3DD
+ myc-CDH1
myc-CDH1
HA-MAD2L2
D.
input (2%)
IP: myc
myc CDH1 only
HA-MAD2L2
WT - 3DD
myc CDH1 only
+ myc-CDH1
HA-MAD2L2
WT 3DD
HA-MAD2L2
WT 2DD
E.
input (2%)
IP: HA
HA-MAD2L2
WT A135D
+ mRFP-CDH1
mRFP-CDH1
+ YFP-CDH1
YFP-CDH1 (long)
YFP-CDH1 (short)
0.4
-75
+ myc-CDH1
myc-CDH1 (long)
myc-CDH1 (short)
0.55
G.
input (2%)
IP: GFP
HA-CDH1
WT F48A
+ YFP-MAD2L2
HA-CDH1
YFP-MAD2L2
-90
H.
IP: HA
CDC27
HA-CDH1
J.
293
myc-MAD2L2
myc-MAD2L2 R124A
myc-MAD2L2 R124A
myc-MAD2L2
CDH1
myc-MAD2L2
actin
-40
I.
IP: HA
IP: MYC
IP: GFP TRAP A
IP: CDH1
α mRFP
α CDC27
α HA
α GFP
α CDH1
IP input

Figure S2

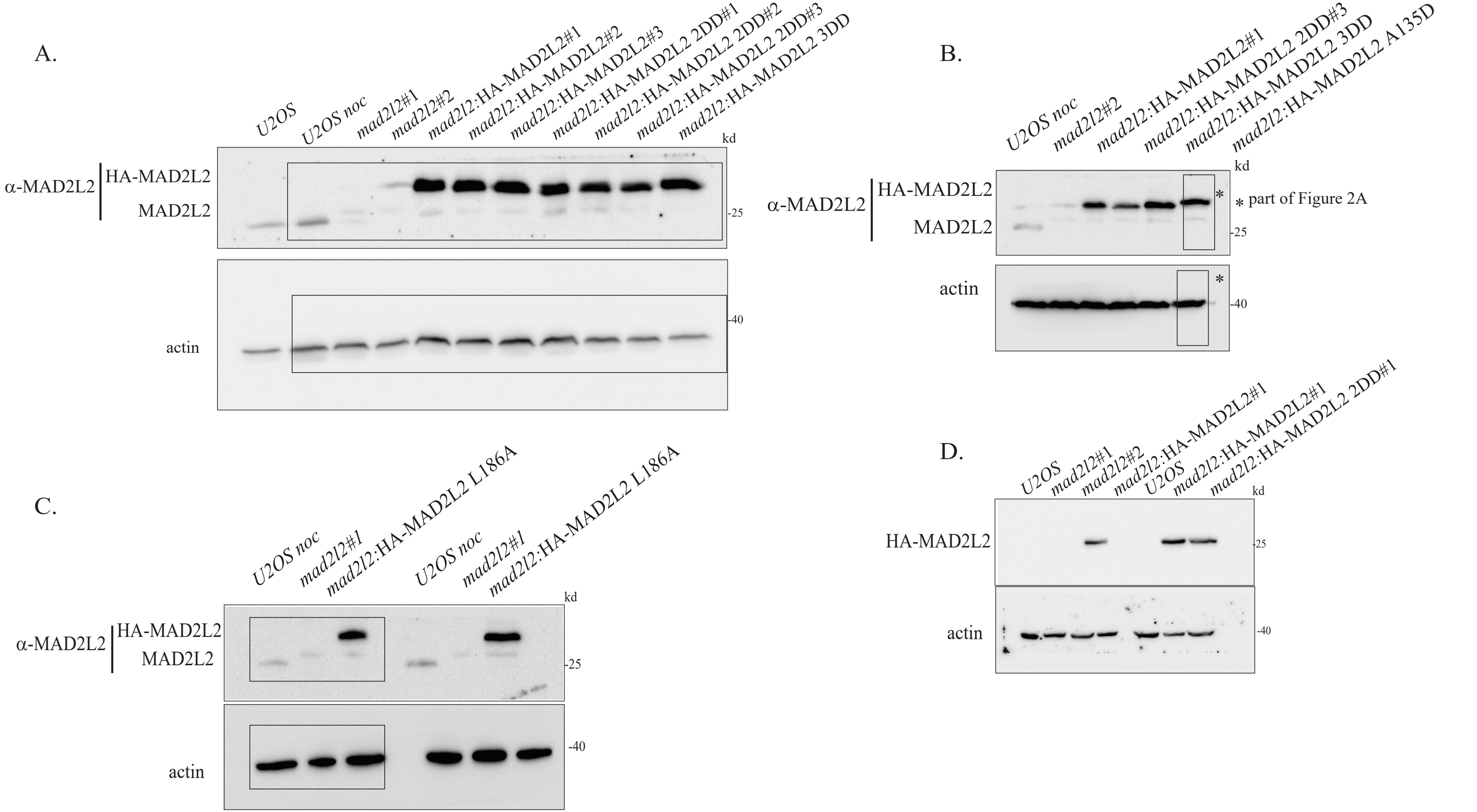
A.
U2OS
U2OS noc
mad2l2#1
mad2l2#2
mad2l2:HA-MAD2L2#1
mad2l2:HA-MAD2L2#2
mad2l2:HA-MAD2L2#3
mad2l2:HA-MAD2L2 2DD#1
mad2l2:HA-MAD2L2 2DD#2
mad2l2:HA-MAD2L2 2DD#3
mad2l2:HA-MAD2L2 3DD
kd
α-MAD2L2
HA-MAD2L2
MAD2L2
-25
-40
actin
B.
U2OS noc
mad2l2#2
mad2l2:HA-MAD2L2#1
mad2l2:HA-MAD2L2 2DD#3
mad2l2:HA-MAD2L2 3DD
mad2l2:HA-MAD2L2 A135D
kd
α-MAD2L2
HA-MAD2L2
MAD2L2
* part of Figure 2A
-25
actin
-40
C.
U2OS noc
mad2l2#1
mad2l2:HA-MAD2L2 L186A
U2OS noc
mad2l2#1
mad2l2:HA-MAD2L2 L186A
kd
α-MAD2L2
HA-MAD2L2
MAD2L2
-25
-40
actin
D.
U2OS
mad2l2#1
mad2l2#2
mad2l2:HA-MAD2L2#1
U2OS
mad2l2:HA-MAD2L2#1
mad2l2:HA-MAD2L2 2DD#1
kd
HA-MAD2L2
-25
actin
-40

Figure S3

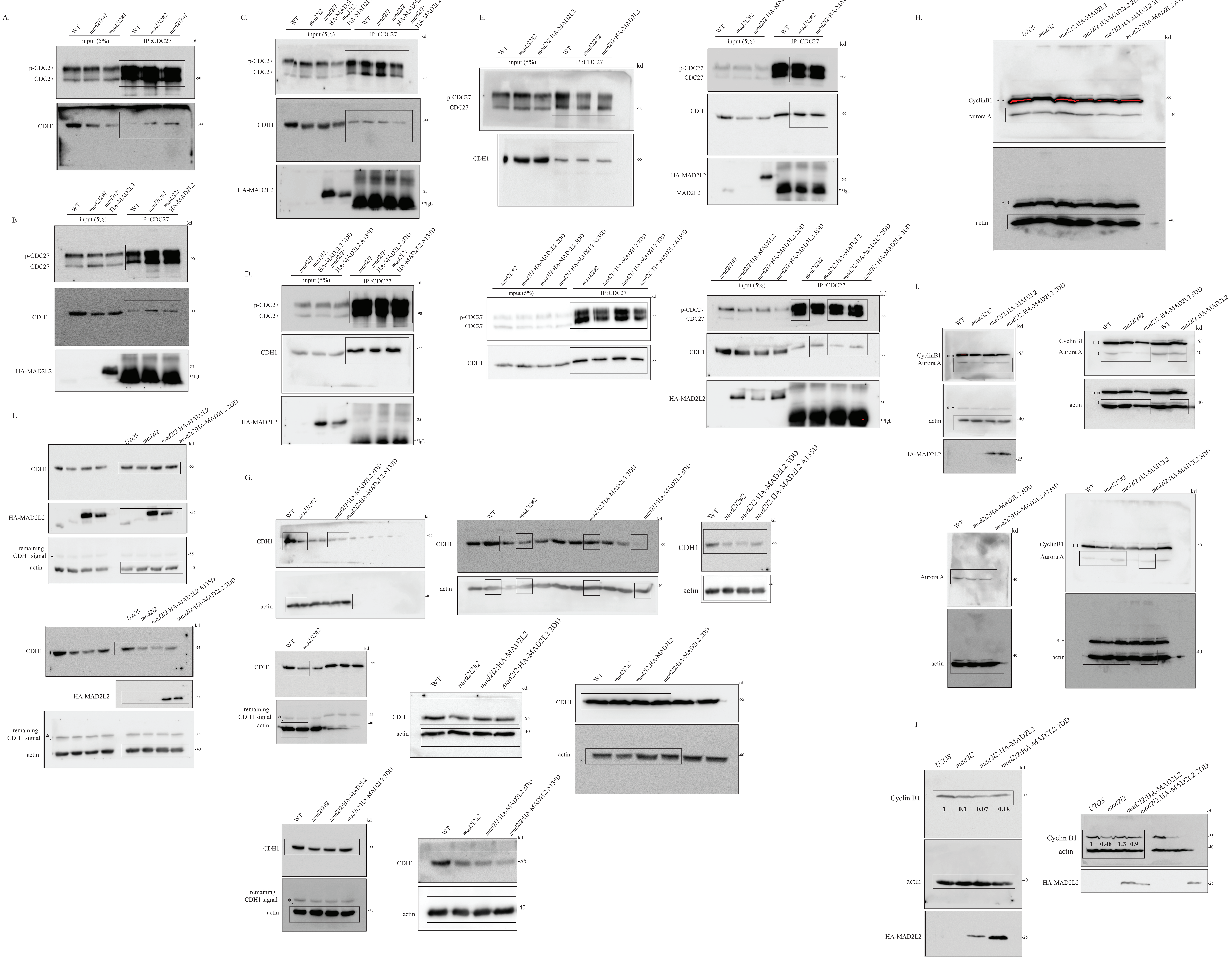
A.
WT
mad2l2#2
mad2l2#1
input (5%)
IP :CDC27
kd
p-CDC27
CDC27
CDH1
B.
mad2l2:HA-MAD2L2
HA-MAD2L2
**IgL
C.
mad2l2
mad2l2:HA-MAD2L2 2DD
D.
mad2l2:HA-MAD2L2 3DD
mad2l2:HA-MAD2L2 A135D
E.
MAD2L2
F.
U2OS
remaining CDH1 signal
actin
G.
H.
CyclinB1
Aurora A
I.
J.
Cyclin B1
1
0.1
0.07
0.18
0.46
1.3
0.9
-90
-55
-40
-25

Supplementary Figure legends

S1. **Monomeric MAD2L2 can bind to CDH1.** (B) Original blot of Figure 1B. (C) Original blot of Figure 1C. (D) The additional blots contributing to the analysis shown in Figure 1D. (E) HA-MAD2L2 A135D mutant displayed reduced binding CDH1. Left panel original blot of Figure 1E. Middle and right panel present reduced binding CDH1 to YFP-CDH1 and myc-CDH1, respectively. HEK293 cells were co-transfected with the indicated plasmids and α-HA IP was performed to assess MAD2L2-CDH1 binding to the HA-MAD2L2 A135D mutant. (G) Original blot of Figure 1G. (H) Original blot of Figure 1H. (I) control IP's of presenting no cross reactivity. (J) Myc-R124A expression affects the expression levels CDH1.HEK293 cells were transfected as indicated. Reduces protein levels of endogenous CDH1 and myc were detected, when compared to myc-MA2DL2 transfected cells.

S2. Monomeric MAD2L2 can regulate mitotic entry. (A-C) Original blot of Figure 1B. Clones of the U2OS CRISPR/Cas9 knockout *mad2l2* cell line, and stably complemented *mad2l2*#1 cell-line with different HA-tagged MAD2L2 mutants, as indicated. (D) Additional blots presenting U2OS CRISPR/Cas9 and complemented cell lines.

S3. Monomeric MAD2L2 inhibits APC/C activation. (A-D) Original blots of the presented IP's. (E) The additional blots contributing to quantification of the relative amount of CDH1 bound to CDC27, in the indicated cell lines. (F) Original blots of the of the relative amount of CDH1 in the indicated cell lines. (G) The additional blots contributing to quantification of the relative amount of CDH1 in the indicated cell lines. (H) Original blots of the of the relative amount of Aurora A in the indicated cell lines. (I) The additional blots contributing to quantification of the relative amount of Aurora A in the indicated cell lines. (J) Premature binding of CDH1 to the APC/C, during nocodazole arrest, leads to reduction in Cyclin B1 levels. Endogenous levels of Cyclin B1 were monitored in the indicated cell lines after 16h of nocodazole treatment. Lack of MAD2L2, or the presence of MAD2L2 without the ability to bind CDH1, leads to premature Cyclin B1 degradation.
